# A Genome-Wide Association Study for Clinical Mastitis in First Parity US Holstein Cows Using Single-Step Approach and Genomic Matrix Re-Weighting Procedure

**DOI:** 10.1371/journal.pone.0114919

**Published:** 2015-02-06

**Authors:** Francesco Tiezzi, Kristen L. Parker-Gaddis, John B. Cole, John S. Clay, Christian Maltecca

**Affiliations:** 1 Department of Animal Science, North Carolina State University, Raleigh, NC, 27695, United States of America; 2 Animal Genomics and Improvement Laboratory, Agricultural Research Service, USDA, Beltsville, MD, 20705–2350, United States of America; 3 Dairy Records Management Systems, Raleigh, NC, 27603, United States of America

## Abstract

Clinical mastitis (CM) is one of the health disorders with large impacts on dairy farming profitability and animal welfare. The objective of this study was to perform a genome-wide association study (GWAS) for CM in first-lactation Holstein. Producer-recorded mastitis event information for 103,585 first-lactation cows were used, together with genotype information on 1,361 bulls from the Illumina BovineSNP50 BeadChip. Single-step genomic-BLUP methodology was used to incorporate genomic data into a threshold-liability model. Association analysis confirmed that CM follows a highly polygenic mode of inheritance. However, 10-adjacent-SNP windows showed that regions on chromosomes 2, 14 and 20 have impacts on genetic variation for CM. Some of the genes located on chromosome 14 (*LY6K*, *LY6D*, *LYNX1*, *LYPD2*, *SLURP1*, *PSCA*) are part of the lymphocyte-antigen-6 complex (*LY6*) known for its neutrophil regulation function linked to the major histocompatibility complex. Other genes on chromosome 2 were also involved in regulating immune response (*IFIH1*, *LY75*, and *DPP4*), or are themselves regulated in the presence of specific pathogens (*ITGB6*, *NR4A2*). Other genes annotated on chromosome 20 are involved in mammary gland metabolism (*GHR*, *OXCT1*), antibody production and phagocytosis of bacterial cells (*C6*, *C7*, *C9*, *C1QTNF3*), tumor suppression (*DAB2*), involution of mammary epithelium (*OSMR*) and cytokine regulation (*PRLR*). DAVID enrichment analysis revealed 5 KEGG pathways. The JAK-STAT signaling pathway (cell proliferation and apoptosis) and the ‘Cytokine-cytokine receptor interaction’ (cytokine and interleukines response to infectious agents) are co-regulated and linked to the ‘ABC transporters’ pathway also found here. Gene network analysis performed using GeneMania revealed a co-expression network where 665 interactions existed among 145 of the genes reported above. Clinical mastitis is a complex trait and the different genes regulating immune response are known to be pathogen-specific. Despite the lack of information in this study, candidate QTL for CM were identified in the US Holstein population.

## Introduction

Clinical mastitis (CM) is a common infectious disease in dairy cows. It is usually defined as an inflammation of the mammary gland resulting from the introduction and multiplication of pathogenic microorganisms, and has been shown to affect farm profitability and animal welfare. Furthermore mastitis brings strong concern about the high risk of culling for diseased animals [1] as well as the delivery of antibiotic residues in milk and the environment due to high frequency of veterinary treatments [2]. A variety of factors impact the susceptibility to mastitis in dairy cows, but selection can be used to limit the frequency of this disease [3]. Examples of direct selection against clinical mastitis through selection indeces can be found in Scandinavian countries, where genetic progress over the last 20 years has shown a positive trend [4,5]. Besides, selection for correlated traits, such as somatic cell score, has been proven to be a valid alternative [6].

The discovery of genomic regions with quantitative impacts on a trait is of essential importance to understand its genetic architecture, and can be used in the design of breeding schemes to increase the frequency of favorable alleles in the population. This is of particular importance when traits have low heritability and there are difficulties in the routine recording of phenotypes, such as resistance to diseases.

The genomic dissection of clinical mastitis is not trivial: etiology depends on a variety of microorganisms (bacteria, fungi, yeasts, and algae can induce mastitis), udder infection can follow different patterns across time (mastitis can be subclinical with a duration of weeks or months, but can be also peracute and lead the cow to death in few days), and pathology can show different levels of intensity according to the individual’s ability to react to pathogens (see Rinaldi et al. [7] for an extensive review). Moreover, resistance to mastitis can be considered as a complex trait, which is likely controlled by many genes with small effects, rather than by few genes with large effect [8].

Health event data voluntarily collected by farmers provide a wealth of information suitable for large-scale breeding value estimation [9]. Unfortunately, these data do not provide any information about the level and duration of exposure to pathogens for every single cow [10], and subclinical infections are often difficult to detect and commonly go unrecorded [3].

These peculiarities in the phenotypic and genetic dissection of CM did not prevent the discovery of associations of this trait with regions of relevant impact across the genome. Several QTL have been found to affect resistance to CM in dairy cattle populations [11–14], although to our knowledge no study has been conducted in the US Holstein population. Detilleux [15] reviewed several studies on potential candidate genes for clinical mastitis, and indicated that genes that are part of the major histocompatibility complex and those linked with neutrophil regulation play major roles in susceptibility to the disease.

Information about regions of the genome involved in resistance to CM currently is lacking in US Holstein, one of the largest dairy cattle populations in the world. The objective of this study was to perform a genome-wide association study for clinical mastitis using producer-recorded health data collected on US dairy cows to identify regions of the genome associated with occurrence of clinical mastitis.

## Materials and Methods

No animal care approval was required for the present manuscript because all records came from field data. The health events for mastitis on first parity cows were extracted from voluntary producer-recorded health event data from Dairy Records Management Systems (Raleigh, NC) as described by [Parker-Gaddis] et al. [16]. Lactation incidence rate was available for 103,585 first-lactation daughters of 10,934 sires, reared in 752 herds. Genotypes were obtained using the Illumina BovineSNP50 BeadChip (Illumina, Inc., San Diego, CA, USA) and included information for 1,361 Holstein bulls. Only genotypes of bulls with at least 5 daughters with phenotype were included in the analysis. Genotypes were edited to eliminate individual markers and individuals with call rate (CR) below 99% and monomorphic markers or markers with minor allele frequency (MAF) below 0.05. Only autosomal markers were used in the analyses. After editing, 39,004 markers were available for analysis. A summary of the data is shown in Table 1.

**Table 1:** Data summary and variance components and heritability estimates using single-step genomic-BLUP methodology.

Number of records	103,585
Number of sires with phenotyped daughters	10,934
Number of sires with genotype	1,361
Number of year-season classes	58
Number of herd-years	3,198
Incidence of disease (%)	10.91
Number of SNPs after editing	39,004
Chromosomes	1–29
Sire additive genetic variance^a^	0.037 0.024 ^(0.026 to 0.047)^
Herd-year variance^a^	0.496 0.006 ^(0.448 to 0.543)^
Residual variance^b^	1
Heritability^a^	0.095 0.014 ^(0.068 to 0.122)^

^a^Posterior mean, posterior standard deviation and 95% Highest Probability Density Intervals are reported.

^b^Residual variance was fixed to 1 in the threshold-liability model.

The association study was performed using the single-step genomic-BLUP approach (ssGBLUP [17–19]). This method was already employed for GWAS by Dikmen et al. [20] and Wang et al. [21]. The model considers additive genetic relationships between the individuals, combining pedigree and genomic information into the **H** matrix [17,22], the inverse of which is constructed by blending the inverse of the SNP-derived genomic matrix (**G**) and the pedigree numerator relationship matrix (**A**) following:

$$H^{-1}=A^{-1}+\left[\begin{array}{cc} 0 & 0\\ 0 & G^{-1}-A_{22}^{-1} \end{array}\right]$$

where **A** ^**-1**^_**22**_ is the inverse of the numerator relationship matrix for the genotyped individuals. In the present study, the **G** matrix was constructed weighting each marker contribution by its expected variance [23]

G=ZDZ'

where **D** is a diagonal matrix with elements containing the inverse of the expected marker variance $D_{ii}=\frac{1}{m\left[2p_{i}(1-p_{i})\right]}$, **Z** is the marker incidence matrix containing genotypes (-1, 0 or 1) corrected by allele frequency [23].

The **H** ^**-1**^ matrix was substituted into the mixed model equation, and a threshold-liability model was used to accommodate the binary nature of the trait. The model used for analyzing CM outcomes (0/1) and obtaining solutions for the sire breeding values was the same as in [Parker-Gaddis] et al. [9]

$$\lambda =X\beta +Z_{h}h+Z_{s}s+e$$

where λ represents a vector of unobserved liabilities to clinical mastitis, β is a vector of fixed effects including the overall mean and year-season, **X** is the corresponding incidence matrix for the fixed effects, *h* represents the random herd-year effect assuming *h*~*N*(0,**I**σ^2^_h_), *s* represents the random sire effect where s~*N*(0,**H**σ^2^_s_), with **H** representing the additive relationship matrix that combines pedigree and genomic information, Z_h_ and Z_s_ represent the corresponding incidence matrices for the herd and sire additive genetic random effects, respectively, and *e* represents the random residual, modeled following *N*(0,**I**σ^2^_e_). Variance components and heritability were estimated using the softwares PREGSF90-POSTGSF90-THRGIBBS1F90 version 2.104 [24–25]. A total of 120,000 iterations were run with the first 20,000 discarded as burn-in, thinning every 10 samples. Post-Gibbs analyses were completed using POSTGIBBSF90 version 3.04 [26] and the ‘coda’ package in R [27]. Trace plots were also inspected visually to ensure convergence had been reached. Posterior standard deviations and 95% highest probability density intervals were calculated for each estimate. re

Marker effects (u) were obtained using an iterative process similar to the one described by Wang et al. [28]. Briefly, after solution of the ssGBLUP model genomic breeding values of genotyped individuals (a_g_) were back-solved to obtain marker effects accounting for their shared genomic variance, as described in the formula

$$\text{var}\left[\begin{array}{c} a_{g}\\ u \end{array}\right]=\left[\begin{array}{cc} ZDZ' & ZD'\\ DZ' & D \end{array}\right]\sigma_{u}^{2}$$

Individual marker effects were obtained by solving:

$$u=DZ'G^{-1}a_{g}$$

In the first round of the iterative process the variance absorbed by each marker was obtained as $2p_{i}(1-p_{i})u^{2}$, where p is the frequency of one of the 2 alleles, **Z** was the marker incidence matrix containing genotypes (-1, 0 or 1) corrected by allele frequency and **G** was the genomic relationship matrix. In successive iterations, in order to highlight regions of higher impact on the genetic variation of the trait, a weighted **G** matrix was created, where expected marker contributions were replaced with realized variances, so that elements of **D** were $D_{ii}=\frac{2p_{i}(1-p_{i})u^{2}}{m}$, with u being the marker effect estimated in the previous iteration. New marker effects were obtained considering the weighted **G** matrix in the formula reported above. For detailed description of the iterative re-weighting procedure please see the ‘Scenario 1’ procedure in Wang et al. [28]. The process was repeated 4 times to ensure stability of estimates.

The variance absorbed by 10-SNPs moving windows was successively calculated across the whole genome. We selected the 10 windows explaining the largest amount of genomic variance for gene annotation, gene network and pathway analyses. Based on the starting and ending coordinates of the windows, gene annotations were obtained using the Biomart platform on Ensemble [29] through the ‘Biomart’ R package (http://www.bioconductor.org). A list of genes located in proximity to the windows was used for performing a gene network analysis using the online resource GeneMania [30], and a pathway-enrichment analysis was performed using the Kyoto Encyclopedia of Genes and Genomes (KEGG [31]) and the Database for Annotation, Visualization and Integrated Discovery (DAVID [32,33]). A Manhattan plot was created using the R package ‘ggplot2’[34].

## Results and Discussion

Summary statistics, variance components, and heritability estimates are reported in Table 1. Phenotypic data used here were similar to those reported by [Parker-Gaddis] et al. [9] for first lactation cows, while genotyped sires and available markers were different. Here, only markers with known positions on autosomes (chromosomes 1 through 29) were used, and 1,361 sires with genotype were included in the analysis. The model used here differs from the previous study because it is a single-trait rather than multi-trait model. Heritabilities and variance components were in agreement in both studies. While no other studies were available to compare genomic estimates of h^2^, our estimates were also in agreement with traditional pedigree-BLUP estimates as reported by other authors in the Holstein as well as in other breeds [3,4,35]. Heritabilities for CM appear to be essentially low, in most of the studies being below 0.10, although genetic variability for immune system capabilities appears to be appreciable. As reported by Detilleux et al. [36], some immune-system parameters measured in cows near calving showed heritabilities larger than 0.10. The reason for this increased uncertainty when modeling the genetic background of any disease resistance trait, such as CM, was suggested by Bishop and Wooliams [10], who remarked that there is low information content when categorizing diagnoses into 2 possible outcomes (healthy/diseased). The absence of knowledge of the level of exposure of animals to the disease, and the lack of information about exposure to specific pathogens, further increases uncertainty. The etiology of mastitis results in a wide range of possibilities for the intensity of inflammation, as found by Lavon et al. [37]: while *Escherichia coli* infections lead to acute responses which make the disease easily identifiable, *Staphylococcus aureus* is more likely to cause subclinical infections, which are probably not reported when phenotyping depends on treatment events and this might have repercussion on the association analyses. In fact, there is a lack of understanding of the genetic variation of the trait when using binary variables for resistance to CM, and some causative mutations may not be identified.

**Fig 1:**
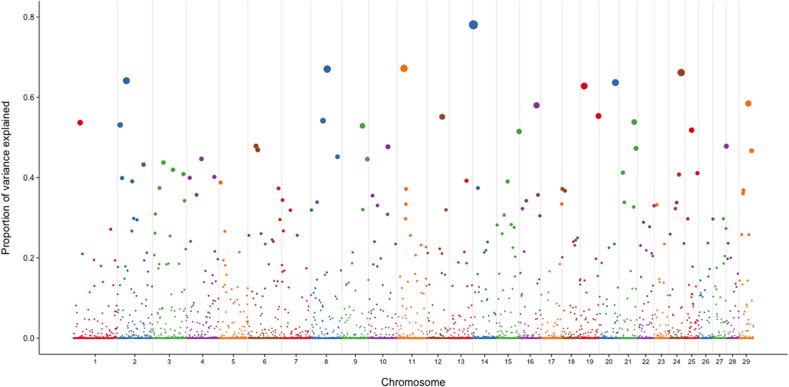
Manhattan plot for the proportion of genetic variance explained by the 10-SNP moving windows. Values on the y-axis sum up to ‘1’.

The present study allowed us to associate clinical mastitis susceptibility to SNP polymorphisms across the genome. The Manhattan plot of marker additive genetic variance explained by 10-SNP moving windows is reported in Fig. 1, and a summary of the 10 windows that explained the largest proportion of variance is provided in Table 2. Clinical mastitis appears to be a moderately polygenic trait, with many regions across the genome contributing to genetic variation. However, there were some regions that appeared to contribute significantly to variation. The re-weighting procedure of the genomic matrix used here shrunk several windows to have adsorbed variance value close to 0. The first 10 windows explained 6.4% of total genomic variance.

**Table 2:** Summary of the 10 windows that explained the most of genetic variance for clinical mastitis in US Holstein dairy cows, with a list of annotated genes in the proximity of each window.

Window	Var, %^a^	Chr	Start, bp	Stop, bp	Genes^b^
2295	0.780	14	2754909	3137184	**LY6K**, ENSBTAG00000037824, **LY6D, LYNX1, LYPD2, SLURP1, THEM6, PSCA**, **BAI1**, **TSNARE1**, ARC
1897	0.671	11	19125116	19644044	VIT, STRN, HEATR5B, GPATCH11, EIF2AK2, SULT6B1
1457	0.671	8	61042106	61507067	MELK, PAX5
3486	0.661	24	46763152	47343727	LOXHD1, ST8SIA5, PIAS2, KATNAL2, HDHD2, IER3IP1
306	0.640	2	30262141	41089812	SCN1A, TTX21B, GALNT3, CSRNP3, SCN2A, SCN3A, SLC38A11, COBLL1, ENSBTAG00000047880, GRB14, KCNH7, GCA, **IFIH1**, FAP, GCG, **DPP4**, SLC4A10, TBR1, PSMD14, TANK, RBMS1, **ITGB6**, PLA2R1, **LY75**, CD302, MARCH7, BAZ2B, WDSUB1, TANC1, DAPL1, PKP4, CCDC148, UPP2, ACVR1C, CYTIP, ERMN, GALNT5, ACVR1, ENSBTAG00000032405, GPD2, **NR4A2**, FIGN
3109	0.637	20	32174117	61609342	**GHR**, FBXO4, C5orf51, **OXCT1**, PLCXD3, **C6**, **C7**, **C9**, MROH2B, **CARD6**, RPL37, PRKAA1, TTC33, PTGER4, **DAB2**, FYB, RICTOR, **OSMR**, LIFR, EGFLAM, GDNF, WDR70, NUP155, C5orf42, NIPBL, ENSBTAG00000047208, SLC1A3, RANBP3L, NADK2, SKP2, LMBRD2, UGT3A1, CAPSL, IL7R, SPEF2, **PRLR**, AGXT2, DNAJC21, BRIX1, RAD1, TTC23L, RAI14, **C1QTNF3**, AMACR, SLC45A2, ADAMTS12, TARS, NPR3, SUB1, ZFR, MTMR12, GOLPH3, PDZD2, ENSBTAG00000038985, C20H5ORF22, DROSHA, CDH6, CDH9, ENSBTAG00000048105, ENSBTAG00000045813, CDH10, CDH12, CDH18, MYO10, FAM134B, ZNF622, MARCH11, FBXL7, ANKH, FAM105B, MGC143209, TRIO, DNAH5, RXFP3, ENSBTAG00000012971, ENSBTAG00000005491
2930	0.628	19	15568242	15997977	TMEM132E
3871	0.584	29	34091321	34723917	OPCML
2657	0.580	16	68069139	68513565	HMCN1, SMIM20
3012	0.553	19	61806709	62390766	MAP2K6, ABAC5, ABAC10, ABAC6, ABAC9, MGC134105, FAM20A, PRKAR1A, ARSG

^a^Single-step genomic-BLUP was used to obtain marker effects.

^b^Genes linked to clinical mastitis are in bold face. Any genes with start and stop positions within or across the window were considered.

In the present study, regions of higher impact on the trait were located in chromosomes 14 (from 2,574,909 to 3,137,184 bp), 11 (from 19,125,116 to 19,644,044 bp), 8 (from 61,042,106 to 61,507,067 bp), 24 (from 46,763,152 to 47,343,727 bp), 2 (from 30,262,141 to 41,089,812 bp), 20 (from 32,174,117 to 61,609,342 bp), 19 (from 15,568,242 to 15,997,977 bp and from 61,806,709 to 68,513,565 bp), 29 (from 34,091,321 to 34,723,917 bp), 16 (from 68,069,139 to 68,513,565 bp). In Danish Holstein, Lund et al. [12] found QTLs on chromosomes 5 (57.8 and 23.8 cM), 6 (116.9 cM), 9 (75.6 and 10.1 cM), 15 (13.7 cM) and 26 (53.4 cM) while Sorensen et al. [38] found QTL on chromosomes 5 (97.5 cM), 9 (4.4, 13.6 and 15.3 cM) and 15 (103.8 and 112.0 cM). In US Holstein, no GWAS has been reported, but Youngerman et al. [39] and Galvao et al. [40] found association between CM and interleukin-8 receptors both located on chromosome 2 at around 90 cM. Sodeland et al. [11], working on Norwegian Red cattle, found several regions affecting occurrence of CM. Some of these were located on chromosomes 2 (from 68 to 112 Mbp), 14 (from 17 to 47 Mbp), 20 (from 31 and 51 Mbp) and 29 (46 Mbp). The authors found that genes encoding for interleukin-8 and the interleukin-8 receptors located on chromosomes 2. In other studies on Scandinavian cattle from different breeds, Holmberg et al. [41] found QTL affecting clinical mastitis on chromosomes 9 (between 130 and 150 cM) and 11 (between 20 and 30 cM); Schulman et al. [13] found SNPs to selectively affect CM on chromosomes 14 at 25 cM and Klungland et al. [11] also identified QTL affecting clinical mastitis on chromosome 14 around 90 cM. Regions affecting CM on chromosome 14 were found in most of the breeds, which is in agreement with the present study.

The markers contained in the 10 most informative windows are reported in S1 Table, while the genes located in correspondence of the 10 windows with the highest variance are reported in Table 2. In window 2295 (chromosome 14, from 2,754,909 to 3,137,184 bp) there were six genes annotated (*LY6K*, *LY6D*, *LYNX1*, *LYPD2*, *SLURP1*, and *PSCA*) that are part of the lymphocyte-antigen-6 complex (*LY6*), which is known for neutrophil regulation function in human and mice, as part of the class III region of the major histocompatibility complex [42,43]. Among those genes LY6D is known to be involved in the first stage of B-cell Leukocyte development in mice [44]. Bahremberg et al. [45] suggested that *PSCA* (prostate stem cell antigen) gene is expressed during hematopoiesis from multipotential stem cells differentiating into leukocyte subpopulations in the peripheral lymphoid tissues, while Adermann et al. [46] reported that *SLURP-1* codes for an amino acid sequence that is similar to the cytotoxins. Moreover, Thuong et al. [47] found *LY6K* to be overexpressed in macrophages extracted from human patients affected by *Mycobacterium tubercolosis*, which is considered an agent of bovine mastitis. On the other hand, *BAI1* seems to be involved in apoptotic cell degradation [48], where it regulates the engulfment of dead cells that are removed prior to infection.

No genes annotated in windows 1897 (chromosome 11, from 19,125,116 to 19,644,044 bp), 1457 (chromosome 8, from 61,042,106 to 61,507,067 bp) and 3486 (chromosome 24, from 46,763,152 to 47,343,727 bp) were found to be related to CM.

Window 306 (chromosome 2, from 30,262,141 to 41,089,812 bp) had five genes know to be related to CM. Kandasamy and Kerr [49] found that *IFIH1* (interferon induced with helicase C, domain 1) was overexpressed in dermal fibroblasts challenged with lipopolysaccharide treatment, concluding that the gene regulates innate immune response. Pimentel et al. [50] found that a SNP located within *IFIH1* increased milk yield and decreased interval from first to last insemination in German Holstein, but reported no association with udder health. *DPP4* was not found to be directly associated with udder health, but is known to interact with cytokines and inter-leukines [51], while integrin beta-6 (*ITGB6*) was found to be differentially expressed in resistant and susceptible lines of sheep challenged with *Staphylococcus* spp. [52]. Lymphocyte antigen 75 (*LY75*) is involved in immune response and inflammation processes [53]. *NR4A2* is not known to regulate udder health, but Moreilhon et al. [54] found that human airway cells infected with *Staphylococcus aureus* induced transcriptional responses of this gene suggesting that it may have a generic function in resistance to bacteria.

Window 3109 located on chromosome 20 and spanning from 32,174,117 to 61,609,342 bp harbored ten genes know to be related to CM. Growth hormone receptor (*GHR*) is known to regulate production traits [55] and other mammary gland phenotypes, as summarized by Ogorevc et al. [56]. Zarrin et al. [57] simulated mastitis in dairy cows with lipopolysaccharide injections and found Succinyl-CoA:3-ketoacid-coenzyme A transferase 1 (*OXCT1*) overexpressed, showing that this gene might regulate mammary gland metabolism and milk synthesis during mastitis infection. Complement components *C6*, *C7*, and *C9* are part of the membrane attack complex [58] and play an important role in immune function, antibody production, inflammation and phagocytosis of bacterial cells. These were found to be associated with CM in the periparturient period [14], and *C7* was also associated with breeding values for somatic cell score [59]. Another complement, C1q and tumor necrosis factor related protein 3 (*C1QTNF3*), was found among the genes overlapping this window and associated with SCS [60]. Likewise, caspase recruitment domain family, member 6 (*CARD6*) is known to be upregulated in cases of *Staphylococcus aureus* infection [61]. *DAB2* is known to be a putative tumor suppressor, and was found to be differentially expressed in mammary glands of cows milked with different patterns [62]. Oncostatin M is a cytokine that is produced post-lactation by the mammary epithelium and is involved in cell death. When its receptor (*OSMR*) was knocked-out in mice the mammary epithelium presented delayed involution [63]. In the case of CM, variants of the *OSMR* gene might be involved in mammary epithelium regeneration after infection and cell apoptosis. Prolactin receptor (*PRLR*) is known to be associated with production traits and somatic cell score [55], and was found to be downregulated in bovine mammary epithelial cells infected with *Staphylococcus aureus* [64], as well as in bovine hepatic tissue following intra-mammary injection of *Escherichia coli* lipopolysaccaride to simulate mastitis infection [65]. Prolactin was found to be significantly increased in udder quarters with high somatic cell count and chronic mastitis [66], and positively correlated with the number of neutrophils in milk, suggesting that prolactin may up-regulate cytokine expression.

The opioid-binding protein/cell adhesion molecule (*OPCML*) found in window 3871 (chromosome 29 from 34,091,321 to 34,723,917 bp). It is a candidate for the suppression of ovarian and broad tumors [67,68].

**Fig 2:**
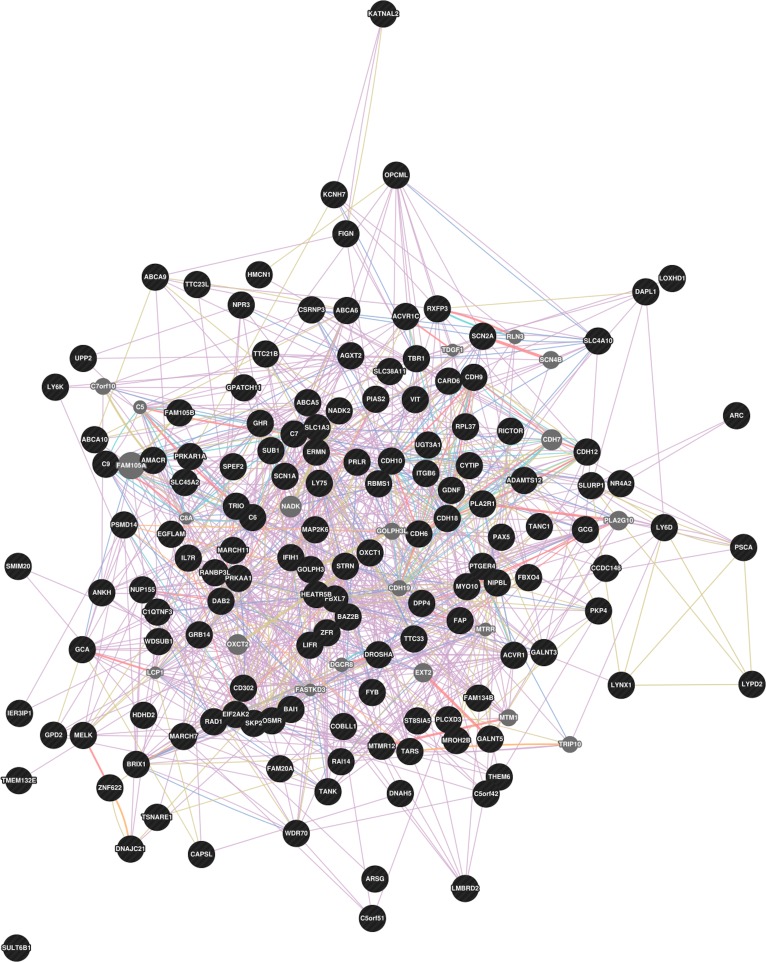
Gene network produced using GeneMANIA. The network consists of 145 genes (circles) connected by 665 interactions (edges).

Gene network analysis performed using GeneMania revealed the dense co-expression network reported in Fig. 2. The network included 145 genes with 665 interactions among them. The number of interactions for each gene in the network is reported in S2 Table. Several genes (*OSMR*, *LY75*, *OXCT1*, *C7*, *GHR*, *C6*, *ITGB6*, *CARD6*, *DAB2*, *DPP4*, *LY6D*, and *PRLR*) presented at least 10 connections, their potential role in determining the resistance to udder infection was discussed above. S1 to S5 Figs. report five functional gene networks highlighted within the general network, respectively ‘Regulation of acute inflammatory response’, ‘Positive regulation of secretion’, ‘Regulation of protein activation cascade’, ‘Regulation of protein activation’, ‘Serine/threonine protein kinase complex’. As expected from a trait with low heritability and highly polygenic architecture, there are a large number of genes involved in the network, with several connections.

Table 3 reports the 5 KEGG pathways identified using genes annotated in the 10 windows with the highest explained variance. The pathways were the ‘Complement and coagulation cascades’, ‘Prion diseases’, ‘ABC transporters’, ‘JAK-STAT signaling pathway’, ‘Cytokine-cytokine receptor interaction’.

**Table 3:** Pathways identified by the Database for Annotation, Visualization and Integrated Discovery (DAVID version 6.7) in the Kyoto Encyclopedia of Genes and Genomes (KEGG).

KEGG entry	Term	Genes count	P-value
Map 04610	Complement and coagulation cascades	7	0.0019
Map 05020	Prior diseases	3	0.0220
Map 02010	ABC transporters	3	0.0320
Map 04630	JAK-STAT signaling pathway	6	0.0321
Map 04060	Cytokine-cytokine receptor interaction	6	0.0120

The JAK-STAT signaling pathway is known for its regulatory role in cell proliferation and apoptosis, responding to the presence of cytokines [69,70]. *JAK2* is considered among the highest-ranking genes for a role in resistance to bovine mastitis [71]. The system works such that the signal is transmitted to the intra-cellular environment through the Janus Kinase (JAK) and signal transducer and activator of transcription (STAT) proteins. This pathway is important in regulating udder response to infection because it controls the persistent accumulation of neutrophils in the bovine mammary gland [72], while in turn JAK also works as a signaling element also for hormones and interleukin receptors [73]. The JAK-STAT pathway is activated during cow lactation [74] and when cattle tissues are challenged with a wide range of pathogen agents, from bacteria such as *Streptococcus uberis* [70], *Escherichia coli* [75], and *Chlamydia trachomatis* [76], to tick-borne protozoan parasites like *Theileria annulata* [77]. The PI3K/Akt pathway can be found within the JAK-STAT signaling pathway: it is overexpressed in lactating cows [78] and in the udder of cows inoculated with *Streptococcus uberis* [72].

The ‘Cytokine-cytokine receptor interaction’ pathway contains the prolactine (*PRLR*), growth hormone (*GHR*) and Inter-leukine 7 (*IL7R*) receptors. The role of cytokines and interleukines in immune response to infectious agents is well known [76,79,80], nonetheless it is recognized that expression of neutrophil chemokines or pro-inflammatory cytokines depends on the kind of pathogen [81–83]. Since our data did not allow differentiation of infections according to the causative pathogen, it is not possible to infer more about genes regulating cytokine expression. The 2 pathways (‘JAK-STAT signaling pathway’ and ‘Cytokine-cytokine receptor interaction’ pathway) are closely linked. Sigl et al. [84] suggested that some JAK-STAT related proteins, as activated by *PRLR*, can regulate balance between growth hormone and milk protein yield. Buitenhuis et al. [75] found both those pathways as being altered in expression in udder tissue of cows challenged with *Escherichia coli*.

The ‘Complement and coagulation cascades pathway’ contained the complement components *C6*, *C7*, and *C9*, as well as the bradikinin receptor (*BDKR*) and serin peptidase inibitors A (*SERPINA1* and *SERPINA5*). Bradikinin is a vasodilator, and high levels of bradikinin may be associated with mastitis infection caused by *Staphylococcus aureus* [85]. Beecher et al. [86] found some variants of *SERPINA1* to be associated with protein yield and fat percentage in Irish Holstein, while Swanson et al. [87] found *SERPINB4* to be slightly upregulated in the mammary gland of cows infected with *Streptococcus uberis*.

Other pathways involving annotated genes were the ‘ABC transporters’ and the ‘Prion diseases’. The former was already found by Naeem et al. [70] to be activated together with the JAK-STAT pathway, and Bionaz et al. [74] found it to be impacted in lactating cows, probably because of its involvement in steroid synthesis and cholesterol regulation.

## Conclusions

The present study demonstrates the complexity of resistance to mastitis pathogens in dairy cattle. Single-step Genomic-BLUP allowed for optimal and simple extraction of genomic information from a population with small fraction of genotyped animals and phenotypes expressed as a categorical variable.

Clinical mastitis was confirmed to be a highly polygenic trait and genetic variance associated with trait was distributed across several regions of the genome. Regions on chromosomes 2, 14, 20, and 29 were found to affect genetic variation for clinical mastitis as well as contain genes with known roles in immune response that are putative QTL. Regions on chromosomes 8, 11, 16, 19, and 24 contained no annotated genes that could be linked to clinical mastitis.

Pathway analysis revealed 5 pathways, 3 of which were know to be involved in immune system regulation, some of them specifically with mastitis resistance in dairy cattle. Pathways and genes found here appear to be differentially expressed in a pathogen-specific manner. Other pathways are known as linked to specific pathogens, but our data did not permit dissection of etiology. The genomic regions identified in this study can be used as predictors of genetic merit for resistance to clinical mastitis in US Holstein dairy cattle.

## Supporting Information

S1 Fig Functional gene network ‘Regulation of acute inflammatory response’ highlighted within the general network. (TIF)

S2 Fig Functional gene network ‘Positive regulation of secretion’ highlighted within the general network. (TIF)

S3 Fig Functional gene network ‘Regulation of protein activation cascade’ highlighted within the general network. (TIF)

S4 Fig Functional gene network ‘Regulation of complement activation’ highlighted within the general network. (TIF)

S5 Fig Functional gene network ‘Serine/threonine protein kinase complex’ highlighted within the general network. (TIF)

S1 Table List of SNPs located in the 10 windows that explained the greatest proportion of marker variance explained. The table reports position (in base pairs), rs (SNP ID from the National Center for Biotechnology Information), SNP name, and the proportion of overall genetic variance explained.
(TIF)

S2 Table List of genes involved in the co-expression network created using GeneMANIA with the respective number of connections for each gene. Names in bold were linked to clinical mastitis based on results of a literature search.
(TIF)

## References

[1] Neerhof HJ, Madsen P, Ducrocq VP, Vollema AR, Jensen J, et al (2000) Relationships between mastitis and functional longevity in Danish Black and White dairy cattle estimated using survival analysis. J Dairy Sci 83: 1064–1071. 10821581 10.3168/jds.S0022-0302(00)74970-8

[2] Heringstad B, Klemetsdal G, Ruane J (2000) Selection for mastitis resistance in dairy cattle: a review with focus on the situation in the Nordic countries. Livest Prod Sci 64: 95–106.

[3] Rupp R, Boichard D (2003) Genetics of resistance to mastitis in dairy cattle. Vet Res 34: 671–688. 14556700 10.1051/vetres:2003020

[4] Heringstad B, Rekaya R, Gianola D, Klemetsdal G, Weigel KA (2003) Genetic change for clinical mastitis in Norwegian Cattle: a threshold model analysis. J Dairy Sci 86: 369–375. 12613880 10.3168/jds.s0022-0302(03)73615-7

[5] Philipsson J, Ral G, Berglund B (1995) Somatic cell count as a selection criterion for mastitis resistance in dairy cattle. Livest Prod Sci 41: 195–200.

[6] CDCB (2014) Trend in somatic cell score for Holstein or Red & White calculated April 2014. Available: https://www.cdcb.us/eval/summary/trend.cfm?R_Menu=HO.s#StartBody. Accessed July 31, 2014.

[7] Rinaldi M, Li RW, Capuco AV (2010) Mastitis associated transcriptomic disruptions in cattle. Vet Immunol Immunopathol 138: 267–279. 10.1016/j.vetimm.2010.10.005 21040982

[8] Pighetti GM, Elliott AA (2011) Gene polymorphisms: The keys for marker assisted selection and unraveling core regulatory pathways for mastitis resistance. J Mammary Gland Biology Neoplasia, 16: 421–432. 10.1007/s10911-011-9238-9 21997401

[9] Parker-Gaddis KL, Cole JB, Clay JS, Maltecca C (2014) Genomic selection for producer-recorded health event data in U.S. dairy cattle. J Dairy Sci 97: 3190–3199. 10.3168/jds.2013-7543 24612803

[10] Bishop SC, Woolliams JA (2010) On the genetic interpretation of disease data. PLoS ONE, 5(1), e8940 10.1371/journal.pone.0008940 20126627 PMC2812510

[11] Klungland H, Sabry A, Heringstad B, Olsen HG, Gomez-Raya L, et al (2001) Quantitative trait loci affecting clinical mastitis and somatic cell count in dairy cattle. Mamm Genome, 12: 837–842. 11845286 10.1007/s00335001-2081-3

[12] Lund MS, Guldbrandtsen B, Buitenhuis AJ, Thomsen B, Bendixen C (2008) Detection of quantitative trait loci in Danish Holstein cattle affecting clinical mastitis, somatic cell score, udder conformation traits, and assessment of associated effects on milk yield. J Dairy Sci 91: 4028–4036. 10.3168/jds.2007-0290 18832229

[13] Schulman NF, Viitala SM, de Koning DJ, Virta J, Mäki-Tanila A, et al (2004) Quantitative trait loci for health traits in Finnish Ayrshire cattle. J Dairy Science 87: 443–449. 14762087 10.3168/jds.S0022-0302(04)73183-5

[14] Sodeland M, Kent MP, Olsen HG, Opsal MA, Svendsen M, et al (2011) Quantitative trait loci for clinical mastitis on chromosomes 2, 6, 14 and 20 in Norwegian Red cattle. Anim Genet 42: 457–465. 10.1111/j.1365-2052.2010.02165.x 21906097

[15] Detilleux JC (2009) Genetic factors affecting susceptibility to udder pathogens. Vet Microbiol 134: 157–164. 10.1016/j.vetmic.2008.09.023 18930606

[16] Parker-Gaddis KL, Cole JB, Clay JS, Maltecca C (2012) Incidence validation and causal relationship analysis of producer-recorded health event data from on-farm computer systems in the U.S. J Dairy Sci 95: 5422–5435. 10.3168/jds.2012-5572 22916949

[17] Aguilar I, Misztal I, Johnson DL, Legarra A, Tsuruta S et al (2010) Hot topic: A unified approach to utilize phenotypic, full pedigree, and genomic information for genetic evaluation of Holstein final score. J Dairy Sci 93: 743–752. 10.3168/jds.2009-2730 20105546

[18] Christensen OF, Lund MS (2010) Genomic prediction when some animals are not genotyped. Genet Sel Evol, 42: 1–8. 10.1186/1297-9686-42-1 20105297 PMC2834608

[19] Misztal I, Aggrey SE, Muir WM (2013) Experiences with a single-step genome evaluation1. Poult Sci 92: 2530–2534. 10.3382/ps.2012-02739 23960138

[20] Dikmen S, Cole JB, Null DJ, Hansen PJ (2013) Genome-wide association mapping for identification of quantitative trait loci for rectal temperature during heat stress in Holstein cattle. PLoS ONE 8: e69202 10.1371/journal.pone.0069202 23935954 PMC3720646

[21] Wang H, Misztal I, Aguilar I, Legarra A, Fernando RL, et al (2014) Genome-wide association mapping including phenotypes from relatives without genotypes in a single-step (ssGWAS) for 6-week body weight in broiler chickens. Front Genet 5: 134 10.3389/fgene.2014.00134 24904635 PMC4033036

[22] Legarra A, Aguilar I, Misztal I (2009) A relationship matrix including full pedigree and genomic information. J Dairy Sci 92: 4656–4663. 10.3168/jds.2009-2061 19700729

[23] VanRaden PM (2008) Efficient methods to compute genomic predictions. J Dairy Sci 91: 4414–4423. 10.3168/jds.2007-0980 18946147

[24] Aguilar I, Misztal I, Tsuruta S, Legarra A, Wang H (2014). PREGSF90–POSTGSF90: Computational Tools for the Implementation of Single-step Genomic Selection and Genome-wide Association with Ungenotyped Individuals in BLUPF90 Programs. In Proc. 10th World Congr. Genet. Appl. Livest. Prod.

[25] Tsuruta S, Misztal I (2006) THRGIBBS1F90 for estimation of variance components with threshold linear models. In Proc. 8th World Congr. Genet. Appl. Livest. Prod. Belo Horizonte, Brazil. Commun. 27–31.

[26] Misztal I, Tsuruta S, Strabel T, Auvray B, Druet T, et al. (2002) BLUPF90 and related programs (BGF90). In Proc. 7th World Congr. Genet. Appl. Livest. Prod. Montpellier, France. 1–2.

[27] Plummer M, Best N, Cowles K, Vines K (2006) CODA: Convergence Diagnosis and Output Analysis for MCMC. R News 6: 7–11.

[28] Wang H, Misztal I, Aguillar I, Legarra A, Muir WM (2012) Genome-wide association mapping including phenotypes from relatives without genotypes. Genet Res 94: 73–83. 10.1017/S0016672312000274 22624567

[29] Flicek P, Ahmed I, Amode MR, Barrell D, Beal K, et al (2013) Ensembl 2013. Nucleic Acids Res 41: D48–D55. 10.1093/nar/gks1236 23203987 PMC3531136

[30] Warde-Farley D, Donaldson SL, Comes O, Zuberi K, Badrawi R, et al (2010) The GeneMANIA prediction server: biological network integration for gene prioritization and predicting gene function. Nucleic Acids Res. 38 Suppl:W214–20. 10.1093/nar/gkq537 20576703 PMC2896186

[31] Kanehisa M, Goto S, Sato Y, Kawashima M, Furumichi M, et al (2014) Data, information, knowledge and principle: back to metabolism in KEGG. Nucleic Acids Res 42: D199–D205 10.1093/nar/gkt1076 24214961 PMC3965122

[32] Huang DW, Sherman BT, Lempicki RA (2009) Bioinformatics enrichment tools: paths toward the comprehensive functional analysis of large gene lists. Nucleic Acids Res 37:1–13. 10.1093/nar/gkn923 19033363 PMC2615629

[33] Huang DW, Sherman BT, Lempicki RA (2009) Systematic and integrative analysis of large gene lists using DAVID Bioinformatics Resources. Nat Protoc 4: 44–57. 10.1038/nprot.2008.211 19131956

[34] Wickham H (2009) ggplot2: Elegant Graphics for Data Analysis Springer, New York, NY 10.14219/jada.archive.2009.0034

[35] Mrode R, Pritchard T, Coffey M, Wall E (2012) Joint estimation of genetic parameters for test-day somatic cell count and mastitis in the United Kingdom. J Dairy Sci 95: 4618–4628. 10.3168/jds.2011-4971 22818477

[36] Detilleux JC, Koehler KJ, Freeman AE, Kehrli ME, Kelley DH (1994) Immunological Parameters of Periparturient Holstein Cattle: Genetic Variation. J Dairy Sci 77: 2640–2650. 7814734 10.3168/jds.S0022-0302(94)77205-2

[37] Lavon Y, Leitner G, Moallem U, Klipper E, Voet H, et al (2011) Immediate and carryover effects of Gram-negative and Gram-positive toxin-induced mastitis on follicular function in dairy cows. Theriogenology 76: 942–953. 10.1016/j.theriogenology.2011.05.001 21705051

[38] Sørensen LP, Guldbrandtsen B, Thomasen JR, Lund MS (2008). Pathogen-specific effects of quantitative trait loci affecting clinical mastitis and somatic cell count in Danish Holstein cattle. J Dairy Sci 91: 2493–2500. 10.3168/jds.2007-0583 18487673

[39] Youngerman SM, Saxton AM, Oliver SP, Pighetti GM (2004) Association of CXCR2 polymorphisms with subclinical and clinical mastitis in dairy cattle. J Dairy Science 87: 2442–2448. 15328266 10.3168/jds.S0022-0302(04)73367-6

[40] Galvao KN, Pighetti GM, Cheong SH, Nydam DV, Gilbert RO (2011) Association between interleukin-8 receptor-α (CXCR1) polymorphism and disease incidence, production, reproduction, and survival in Holstein cows. J Dairy Sci 94: 2083–2091. 10.3168/jds.2010-3636 21426999

[41] Holmbeg M, Andersson-Eklund L (2004) Quantitative trait loci affecting health traits in Swedish dairy cattle. J Dairy Sci 87: 2653–2659. 15328290 10.3168/jds.s0022-0302(04)73391-3

[42] Horton R, Wilming L, Rand V, Lovering RC, Bruford EA et al (2004) Gene map of the extended human MHC. Nature Rev Genetics 5: 889–899. 15573121 10.1038/nrg1489

[43] Mallya M, Campbell RD, Aguado B (2002) Transcriptional analysis of a novel cluster of LY-6 family members in the human and mouse major histocompatibility complex: five genes with many splice forms. Genomics 80: 113–123. 12079290 10.1006/geno.2002.6794

[44] Inlay MA, Bhattacharya D, Sahoo D, Serwold T, Seita J, et al (2009) Ly6d marks the earliest stage of B-cell specification and identifies the branchpoint between B-cell and T-cell development. Genes Dev 23: 2376–2381. 10.1101/gad.1836009 19833765 PMC2764492

[45] Bahrenberg G, Brauers A, Joost HG, Jakse G (2000) Reduced expression of PSCA, a member of the LY-6 family of cell surface antigens, in bladder, esophagus, and stomach tumors. Biochem Biophys Res Comm 275: 783–788. 10973799 10.1006/bbrc.2000.3393

[46] Adermann K, Wattler F, Wattler S, Heine G, Meyer M, et al (1999) Structural and phylogenetic characterization of human SLURP‐1, the first secreted mammalian member of the Ly‐6/uPAR protein superfamily. Protein Sci 8: 810–819. 10211827 10.1110/ps.8.4.810 PMC2144295

[47] Thuong NTT, Dunstan SJ, Chau TTH, Thorsson V, Simmons CP, et al (2008) Identification of tuberculosis susceptibility genes with human macrophage gene expression profiles. PLoS Pathog 4: e1000229 10.1371/journal.ppat.1000229 19057661 PMC2585058

[48] Park D, Tosello-Trampont AC, Elliott MR, Lu M, Haney LB, et al (2007) BAI1 is an engulfment receptor for apoptotic cells upstream of the ELMO/Dock180/Rac module. Nature, 450: 430–434. 17960134 10.1038/nature06329

[49] Kandasamy S, Kerr DE (2012) Genomic analysis of between-cow variation in dermal fibroblast response to lipopolysaccharide. J Dairy Sci 95: 3852–3864. 10.3168/jds.2011-5251 22720940 PMC4235160

[50] Pimentel ECG, Bauersachs S, Tietze M, Simianer H, Tetens J, et al (2011) Exploration of relationships between production and fertility traits in dairy cattle via association studies of SNPs within candidate genes derived by expression profiling. Animal Genet 42: 251–262. 10.1111/j.1365-2052.2010.02148.x 21198698

[51] Lee SU, Ferens W, Davis WC, Hamilton MJ, Park YH, et al (2001) Identity of activation molecule 3 on superantigen-stimulated bovine cells is CD26. Infect Immun 69: 7190–7193. 11598101 10.1128/IAI.69.11.7190-7193.2001 PMC100126

[52] Bonnefont CM, Toufeer M, Caubet C, Foulon E, Tasca C, et al (2011) Transcriptomic analysis of milk somatic cells in mastitis resistant and susceptible sheep upon challenge with Staphylococcus epidermidis and Staphylococcus aureus. BMC Genomics 12: 208 10.1186/1471-2164-12-208 21527017 PMC3096985

[53] Schwerin M, Czernek-Schafer D, Goldammer T, Kata SR, Womack JE, et al (2003) Application of disease-associated differentially expressed genes-Mining for functional candidate genes for mastitis resistance in cattle. Genet Sel Evol 35: S19–S34. 12927078 10.1186/1297-9686-35-S1-S19 PMC3231760

[54] Moreilhon C, Gras D, Hologne C, Bajolet O, Cottrez F, et al (2005) Live Staphylococcus aureus and bacterial soluble factors induce different transcriptional responses in human airway cells. Physiol Genomics 20: 244–255. 15598879 10.1152/physiolgenomics.00135.2004

[55] Meredith BK, Kearney FJ, Finlay EK, Bradley DG, Fahey AG, et al (2012) Genome-wide associations for milk production and somatic cell score in Holstein-Friesian cattle in Ireland. BMC Genetics 13: 21 10.1186/1471-2156-13-21 22449276 PMC3361482

[56] Ogorevc J, Kunej T, Razpet A, Dovc P (2009) Database of cattle candidate genes and genetic markers for milk production and mastitis. Anim Genet 40: 832–851. 10.1111/j.1365-2052.2009.01921.x 19508288 PMC2779988

[57] Zarrin M, Wellnitz O, van Dorland HA, Gross JJ, Bruckmaier RM (2014) Hyperketonemia during lipopolysaccharide-induced mastitis affects systemic and local intramammary metabolism in dairy cows. J Dairy Sci 97: 3531–3541. 10.3168/jds.2013-7480 24679930

[58] Rainard P (2003) The complement in milk and defense of the bovine mammary gland against infections. Vet Res 34: 647–670. 14556699 10.1051/vetres:2003025

[59] He Y, Chu Q, Ma P, Wang Y, Zhang Q, et al (2011) Association of bovine CD4 and STAT5b single nucleotide polymorphisms with somatic cell scores and milk production traits in Chinese Holsteins. J Dairy Res 78: 242–249. 10.1017/S0022029911000148 21435309

[60] Meredith BK, Berry DP, Kearney F, Finlay EK, Fahey AG, et al. (2013) A genome-wide association study for somatic cell score using the Illumina high-density bovine beadchip identifies several novel QTL potentially related to mastitis susceptibility. Front Genet 4. 10.3389/fgene.2013.00229 PMC3818585 24223582

[61] Thacker JD, Balin BJ, Appelt DM, Sassi-Gaha S, Purohit M, et al (2012) NLRP3 inflammasome is a target for development of broad-spectrum anti-infective drugs. Antimicrob Agents Chemother 56: 1921–1930. 10.1128/AAC.06372-11 22290938 PMC3318317

[62] Connor EE, Siferd S, Elsasser TH, Evock-Clover CM, Van Tassell CP, et al (2008) Effects of increased milking frequency on gene expression in the bovine mammary gland. BMC Genomics, 9: 362 10.1186/1471-2164-9-362 18671851 PMC2518935

[63] Stanford JC, Cook RS (2013) Apoptosis and Clearance of the Secretory Mammary Epithelium.

[64] Lara-Zárate L, López-Meza JE, Ochoa-Zarzosa A (2011) Staphylococcus aureus inhibits nuclear factor kappa B activation mediated by prolactin in bovine mammary epithelial cells. Microb Pathog 51: 313–318. 10.1016/j.micpath.2011.07.010 21843629

[65] Jiang L, Sørensen P, Røntved C, Vels L, Ingvartsen KL (2008) Gene expression profiling of liver from dairy cows treated intra-mammary with lipopolysaccharide. BMC Genomics, 9: 443 10.1186/1471-2164-9-443 18816405 PMC2576255

[66] Boutet P, Sulon J, Closset R, Detilleux J, Beckers JF, et al (2007) Prolactin-Induced Activation of Nuclear Factor κ B in Bovine Mammary Epithelial Cells: Role in Chronic Mastitis. J Dairy Sci 90: 155–164. 17183084 10.3168/jds.S0022-0302(07)72617-6

[67] Sellar GC, Watt KP, Rabiasz GJ, Stronach EA, Li L, Miller EP, et al (2003) OPCML at 11q25 is epigenetically inactivated and has tumor-suppressor function in epithelial ovarian cancer. Nat Genet 34: 337–343. 12819783 10.1038/ng1183

[68] Cui Y, Ying Y, van Hasselt A, Ng KM, Yu J, et al (2008) OPCML is a broad tumor suppressor for multiple carcinomas and lymphomas with frequently epigenetic inactivation. PLoS One, 3: e2990 10.1371/journal.pone.0002990 18714356 PMC2500176

[69] Bionaz M, Loor JJ (2008) Gene networks driving bovine milk fat synthesis during the lactation cycle. BMC Genomics, 9: 366 10.1186/1471-2164-9-366 18671863 PMC2547860

[70] Naeem A, Zhong K, Moisá SJ, Drackley JK, Moyes KM, et al (2012) Bioinformatics analysis of microRNA and putative target genes in bovine mammary tissue infected with Streptococcus uberis. J Dairy Sci 95: 6397–6408. 10.3168/jds.2011-5173 22959936

[71] Jiang L, Sørensen P, Thomsen B, Edwards SM, Skarman A, et al (2012) Gene prioritization for livestock diseases by data integration. Physiol Gen 44: 305–317. 10.1152/physiolgenomics.00047.2011 22234994

[72] Boutet P, Boulanger D, Gillet L, Vanderplasschen A, Closset R, et al (2004) Delayed neutrophil apoptosis in bovine subclinical mastitis. J Dairy Science, 87: 4104–4114. 15545372 10.3168/jds.S0022-0302(04)73553-5

[73] Moyes KM, Drackley JK, Morin DE, Bionaz M, Rodriguez-Zas SL, et al (2009) Gene network and pathway analysis of bovine mammary tissue challenged with Streptococcus uberis reveals induction of cell proliferation and inhibition of PPARγ signaling as potential mechanism for the negative relationships between immune response and lipid metabolism. BMC Genomics, 10: 542 10.1186/1471-2164-10-542 19925655 PMC2784807

[74] Bionaz M, Periasamy K, Rodriguez-Zas SL, Everts RE, Lewin HA, et al (2012) Old and new stories: revelations from functional analysis of the bovine mammary transcriptome during the lactation cycle. PloS ONE, 7: e33268 10.1371/journal.pone.0033268 22428004 PMC3299771

[75] Buitenhuis B, Røntved CM, Edwards SM, Ingvartsen KL, Sørensen P (2011) In depth analysis of genes and pathways of the mammary gland involved in the pathogenesis of bovine Escherichia coli-mastitis. BMC Genomics, 12: 130 10.1186/1471-2164-12-130 21352611 PMC3053262

[76] Lad SP, Fukuda EY, Li J, Luis M, Li E (2005) Up-regulation of the JAK/STAT1 signal pathway during Chlamydia trachomatis infection. The J Immunol, 174: 7186–7193. 15905563 10.4049/jimmunol.174.11.7186

[77] Glass EJ, Jensen K (2007) Resistance and susceptibility to a protozoan parasite of cattle gene expression differences in macrophages from different breeds of cattle. Vet Immunol Immunopathol 120: 20–30. 17727964 10.1016/j.vetimm.2007.07.013

[78] Brenaut P, Bangera R, Bevilacqua C, Rebours E, Cebo C, et al (2012) Validation of RNA isolated from milk fat globules to profile mammary epithelial cell expression during lactation and transcriptional response to a bacterial infection. J Dairy Sci 95: 6130–6144. 10.3168/jds.2012-5604 22921620

[79] Griesbeck-Zilch B, Osman M, Kühn C, Schwerin M, Bruckmaier RH, et al (2009) Analysis of key molecules of the innate immune system in mammary epithelial cells isolated from marker-assisted and conventionally selected cattle. J Dairy Sci 92: 4621–4633. 10.3168/jds.2008-1954 19700725

[80] Rambeaud M, Almeida RA, Pighetti GM, Oliver SP (2003) Dynamics of leukocytes and cytokines during experimentally induced Streptococcus uberis mastitis. Veter Immunol Immunopathol 96: 193–205. 14592732 10.1016/j.vetimm.2003.08.008

[81] Lahouassa H, Moussay E, Rainard P, Riollet C (2007) Differential cytokine and chemokine responses of bovine mammary epithelial cells to Staphylococcus aureus and Escherichia coli. Cytokine, 38: 12–21. 17532224 10.1016/j.cyto.2007.04.006

[82] Lee JW, Bannerman DD, Paape MJ, Huang MK, Zhao X (2006) Characterization of cytokine expression in milk somatic cells during intramammary infections with Escherichia coli or Staphylococcus aureus by real-time PCR. Vet Res 37: 219–229. 16472521 10.1051/vetres:2005051

[83] Gutiérrez-Barroso A, Anaya-López JL, Lara-Zárate L, Loeza-Lara PD, López-Meza JE, et al (2008) Prolactin stimulates the internalization of Staphylococcus aureus and modulates the expression of inflammatory response genes in bovine mammary epithelial cells. Veter Immunol and Immunopathol 121: 113–122. 17988748 10.1016/j.vetimm.2007.09.007

[84] Sigl T, Meyer HHD, Wiedemann S (2014) Gene expression analysis of protein synthesis pathways in bovine mammary epithelial cells purified from milk during lactation and short‐term restricted feeding. J Anim Physiol Animal Nutr 98: 84–95. 10.1111/jpn.12039 23402545

[85] Eshraghi HR, Zeitlin IJ, Fitzpatrick JL, Ternent H, Logue D (1999) The release of bradykinin in bovine mastitis. Life Sci 64: 1675–1687. 10328527 10.1016/s0024-3205(99)00105-8

[86] Beecher C, Daly M, Childs S, Berry DP, Magee DA, et al (2010) Polymorphisms in bovine immune genes and their associations with somatic cell count and milk production in dairy cattle. BMC Genetics, 11: 99 10.1186/1471-2156-11-99 21054834 PMC3087511

[87] Swanson KM, Stelwagen K, Dobson J, Henderson HV, Davis SR, et al (2009) Transcriptome profiling of Streptococcus uberis induced mastitis reveals fundamental differences between immune gene expression in the mammary gland and in a primary cell culture model. J Dairy Sci 92: 117–129. 10.3168/jds.2008-1382 19109270

