# Supplementary figures and images for: A Genome-Wide Association Study for Clinical Mastitis in First Parity US Holstein Cows Using Single-Step Approach and Genomic Matrix Re-Weighting Procedure
Source: PLoS One. 2015 Feb 6;10(2):e0114919. doi: 10.1371/journal.pone.0114919 (PMC4319771; doi:10.1371/journal.pone.0114919)

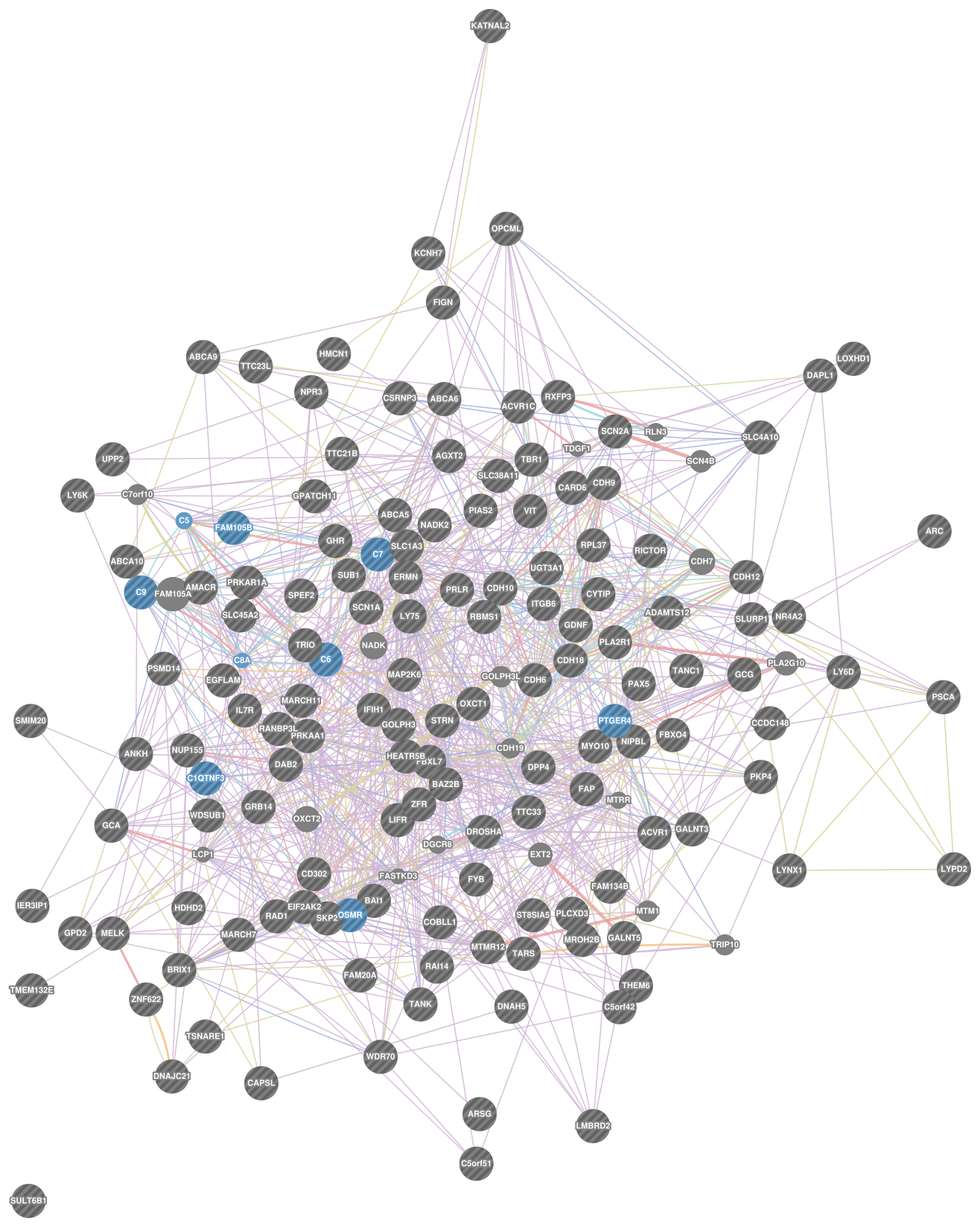

Supplement: S1 Fig — (TIF) [file pone.0114919.s001.tif]

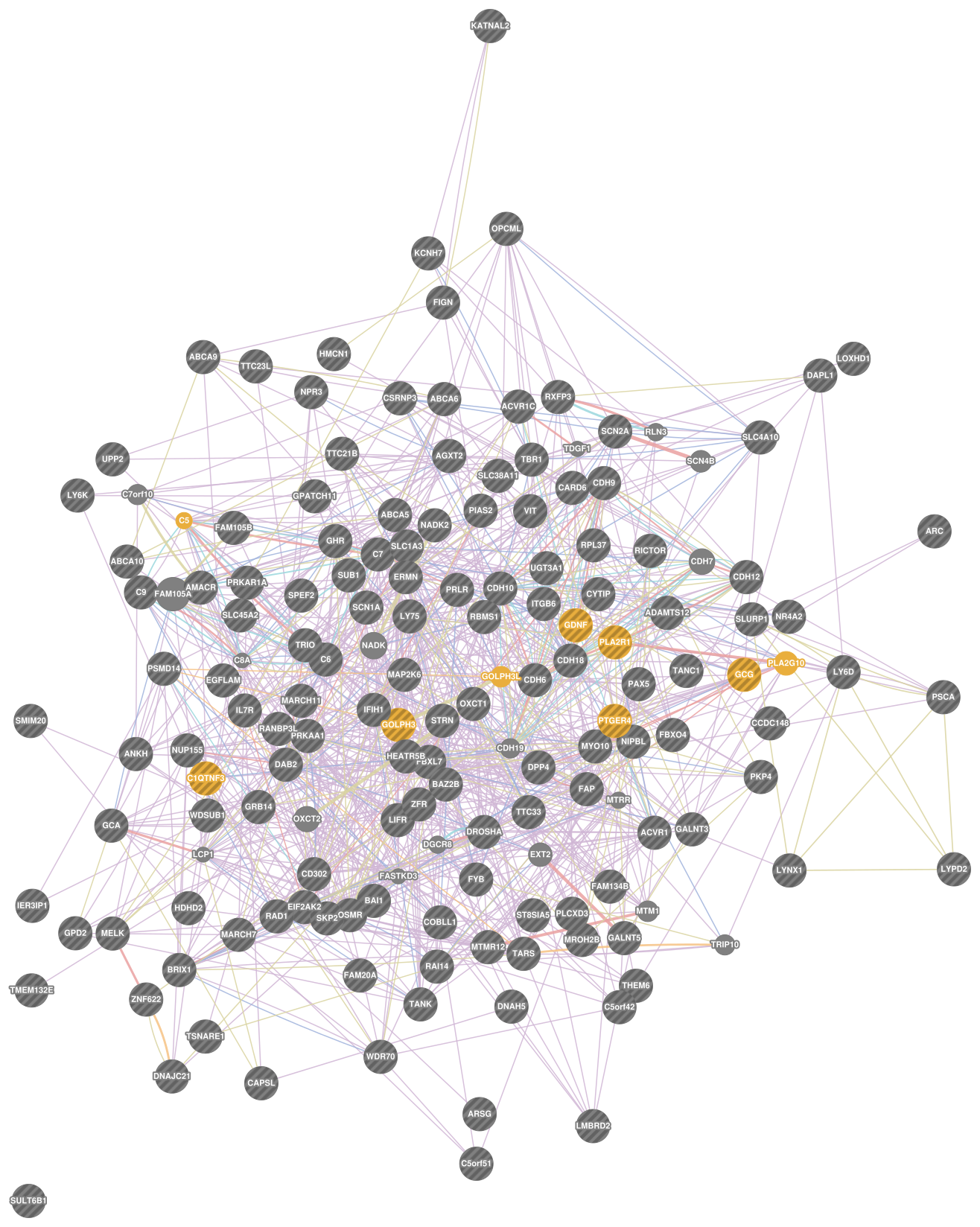

Supplement: S2 Fig — (TIF) [file pone.0114919.s002.tif]

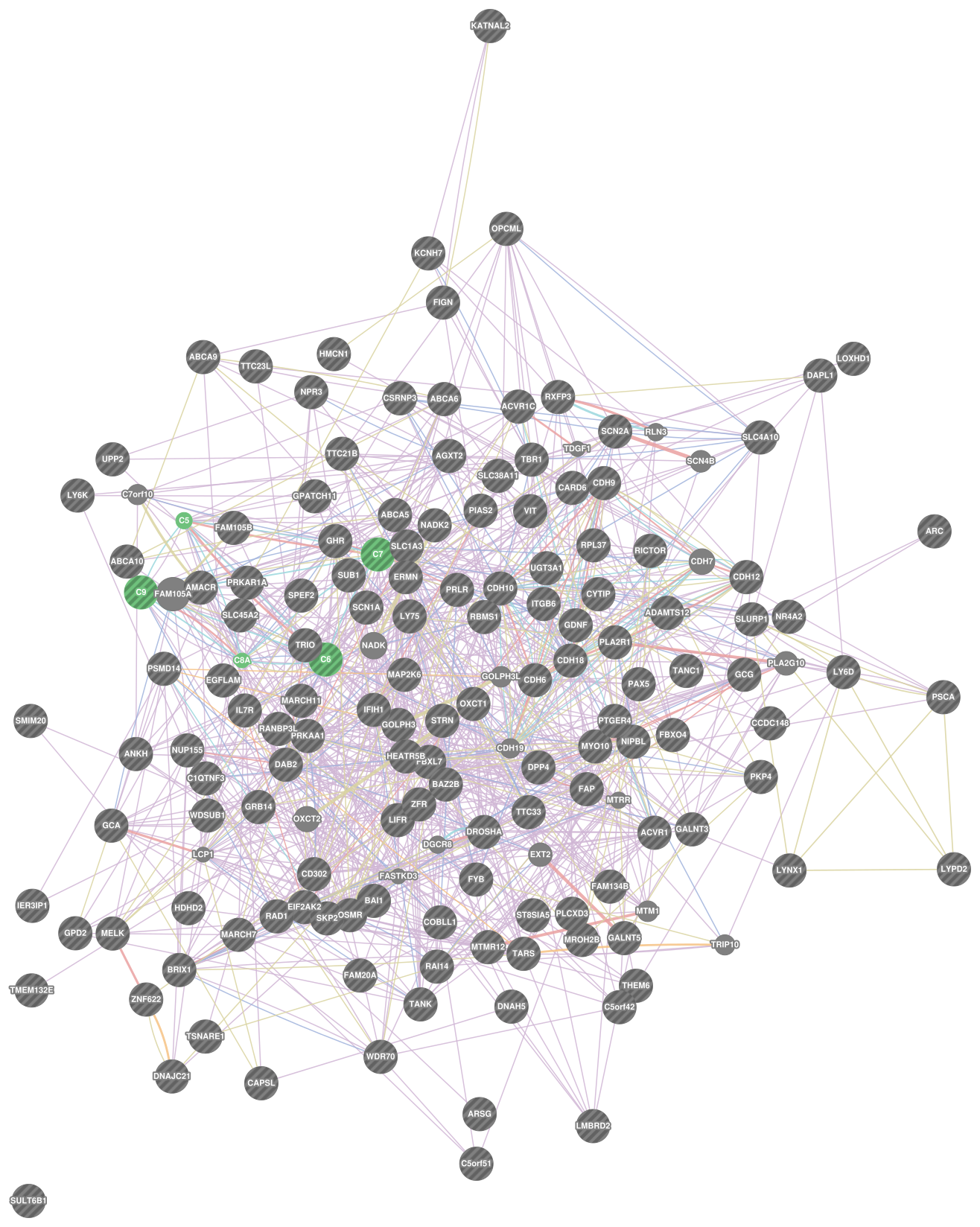

Supplement: S3 Fig — (TIF) [file pone.0114919.s003.tif]

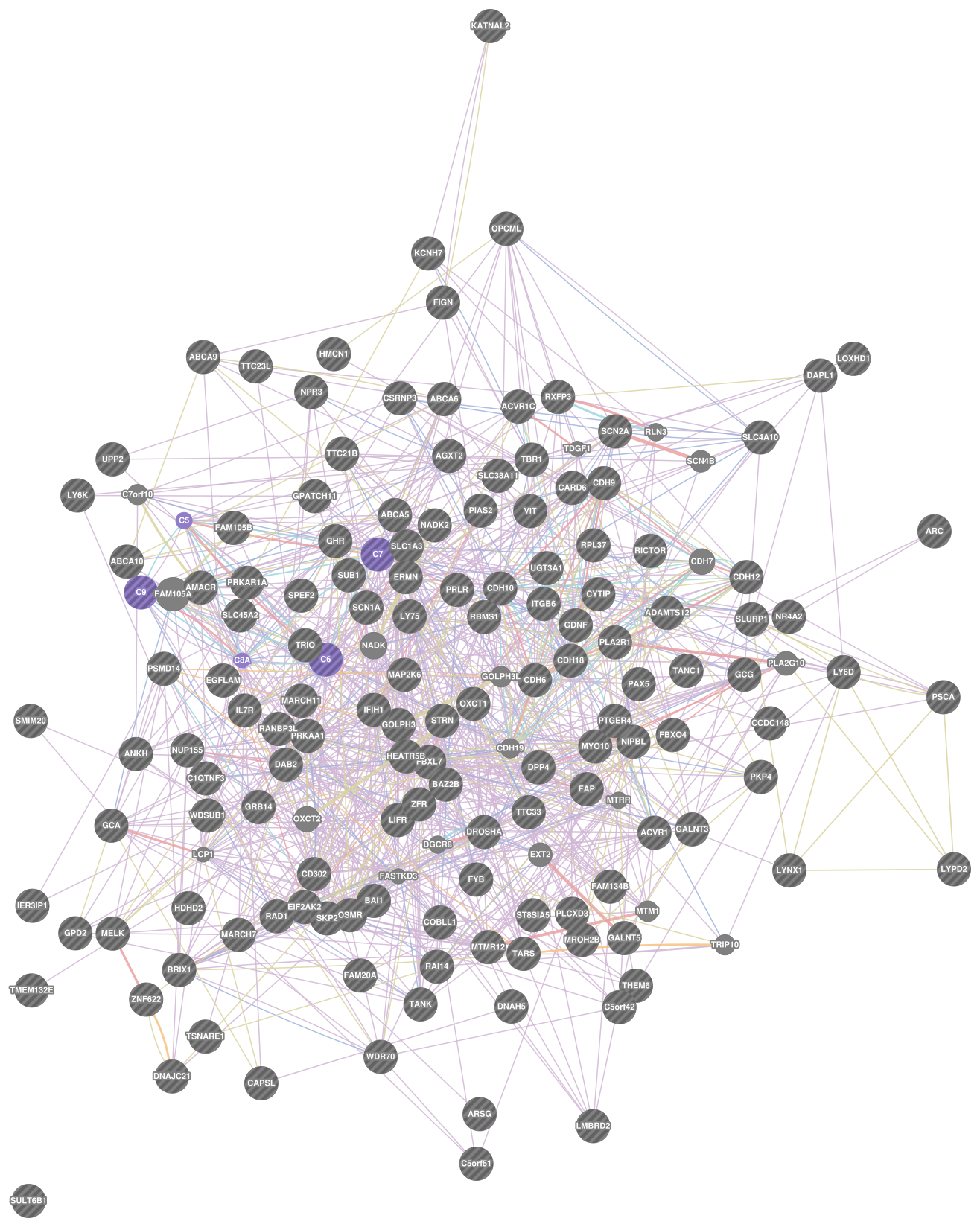

Supplement: S4 Fig — (TIF) [file pone.0114919.s004.tif]

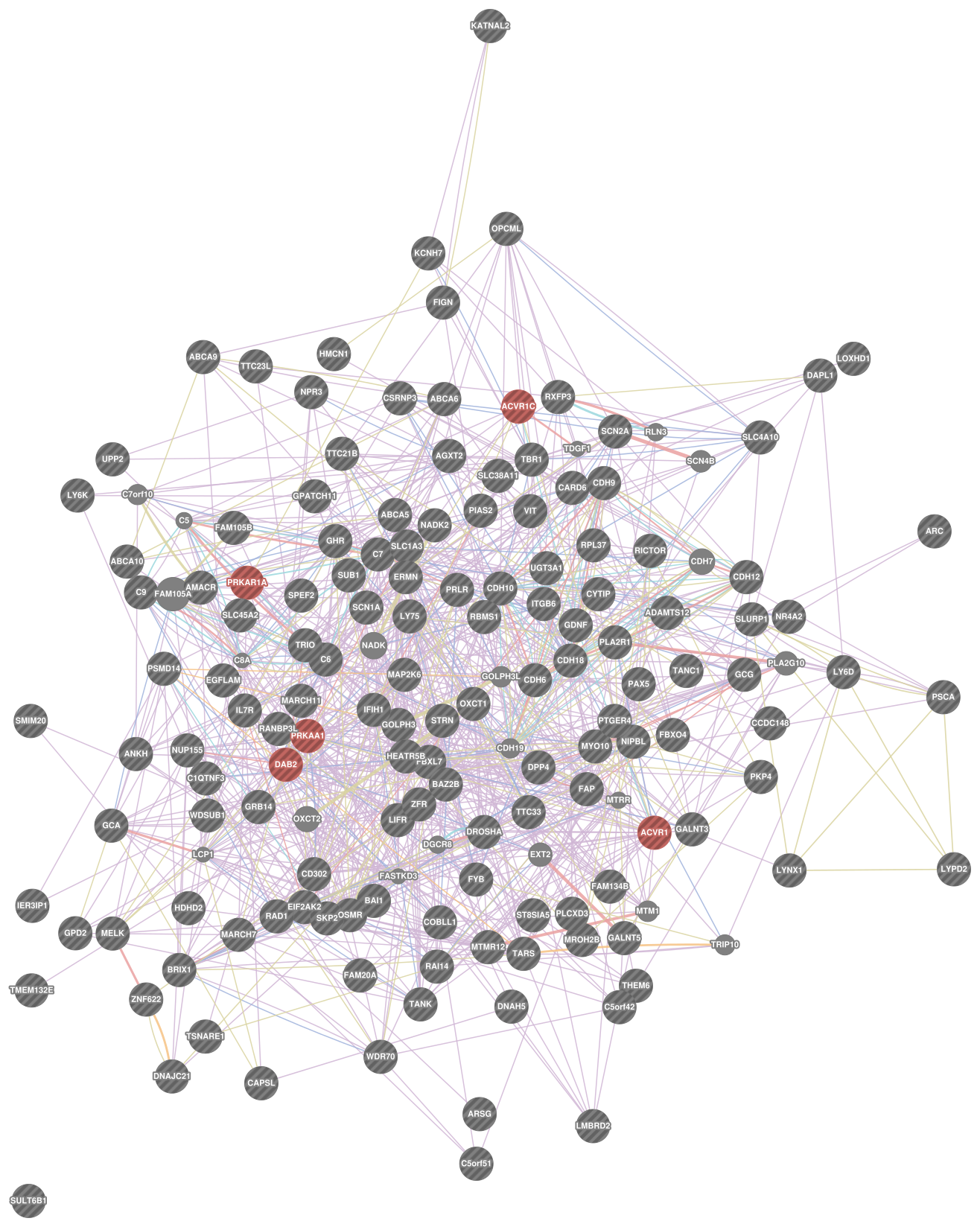

Supplement: S5 Fig — (TIF) [file pone.0114919.s005.tif]

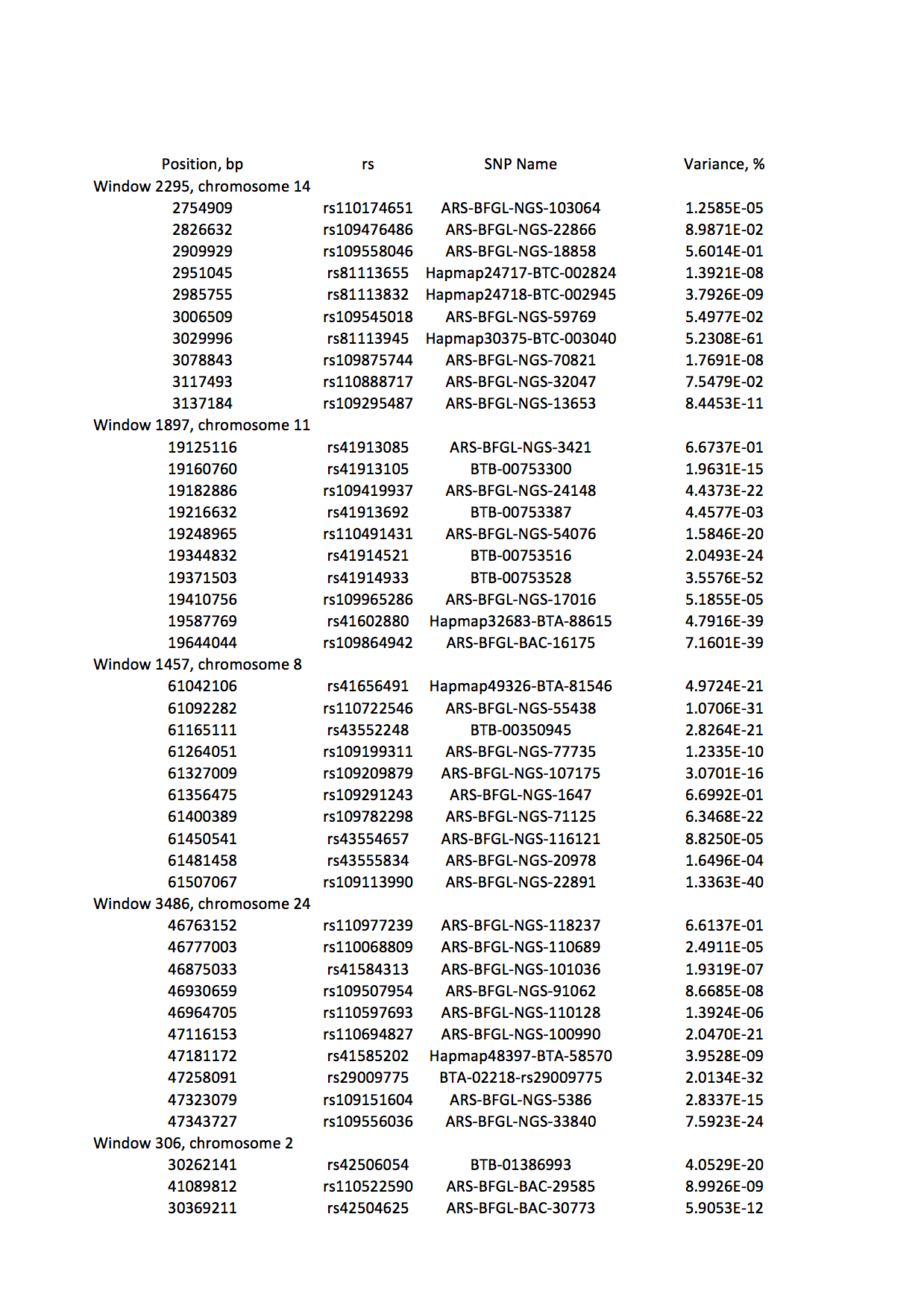

Supplement: S1 Table — The table reports position (in base pairs), rs (SNP ID from the National Center for Biotechnology Information), SNP name, and the proportion of overall genetic variance explained. (TIF) [file pone.0114919.s006.tif]

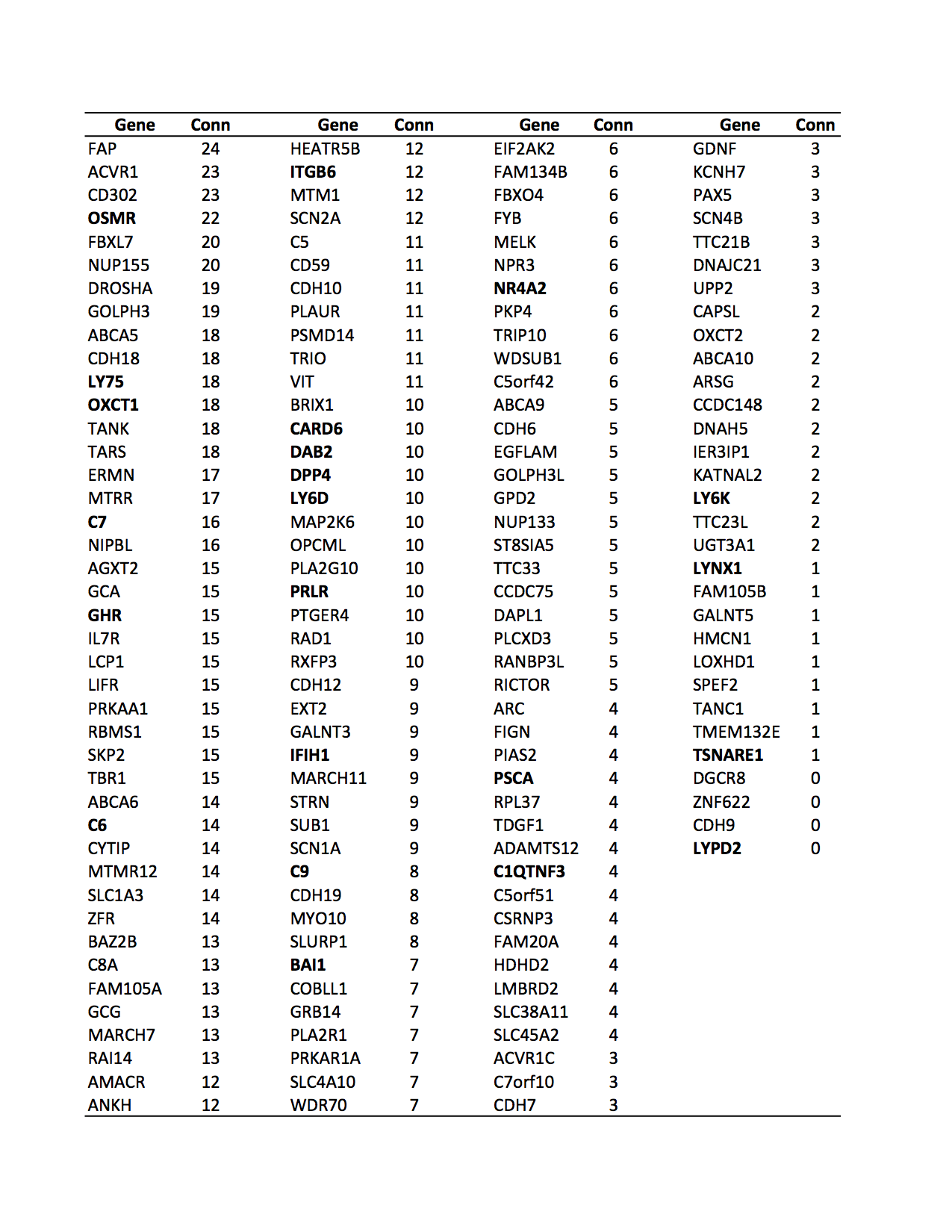

Supplement: S2 Table — Names in bold were linked to clinical mastitis based on results of a literature search. (TIF) [file pone.0114919.s007.tif]
